# Engineering Safety-Oriented Blasting-Induced Seismic Wave Signal Processing: An EMD Endpoint Suppression Method Based on Multi-Scale Feature

**DOI:** 10.3390/s25134194

**Published:** 2025-07-05

**Authors:** Miao Sun, Jing Wu, Yani Lu, Fangda Yu, Hang Zhou

**Affiliations:** 1 School of Civil Engineering & Research Center of Hubei Small Town Development, Hubei Engineering University, Xiaogan 432000, China; ms199405@outlook.com (M.S.); lyn2016@hbeu.edu.cn (Y.L.); yfd202505@outlook.com (F.Y.); 2 Engineering Research Center of Rock-Soil Drilling & Excavation and Protection, Ministry of Education, China University of Geosciences, Wuhan 430074, China; 3 School of Information Engineering, Chang’an University, Xi’an 710064, China; hz200312@outlook.com

**Keywords:** blasting seismic wave, empirical mode decomposition, intrinsic mode function, endpoint effect, hazard control

## Abstract

Blasting-induced seismic waves are typically nonlinear and non-stationary signals. The EMD-Hilbert transform is commonly used for time–frequency analysis of such signals. However, during the empirical mode decomposition (EMD) processing of blasting-induced seismic waves, endpoint effects occur, resulting in varying degrees of divergence in the obtained intrinsic mode function (IMF) components at both ends. The further application of the Hilbert transform to these endpoint-divergent IMFs yield artificial time–frequency analysis results, adversely impacting the assessment of blasting-induced seismic wave hazards. This paper proposes an improved EMD endpoint effect suppression algorithm that considers local endpoint development trends, global time distribution, energy matching, and waveform matching. The method first analyzes global temporal characteristics and endpoint amplitude variations to obtain left and right endpoint extension signal fragment S(*t*)_L_ and S(*t*)_R_. Using these as references, the original signal is divided into “b” equal segments S(*t*)_1_, S(*t*)_2_ … S(*t*)_b_. Energy matching and waveform matching functions are then established to identify signal fragments S(*t*)_i_ and S(*t*)*_j_* that match both the energy and waveform characteristics of S(*t*)_L_ and S(*t*)_R_. Replacing S(*t*)_L_ and S(*t*)*_R_* with S(*t*)_i_ and S(*t*)_j_ effectively suppresses the EMD endpoint effects. To verify the algorithm’s effectiveness in suppressing EMD endpoint effects, comparative studies were conducted using simulated signals to compare the proposed method with mirror extension, polynomial fitting, and extreme value extension methods. Three evaluation metrics were utilized: error standard deviation, correlation coefficient, and computation time. The results demonstrate that the proposed algorithm effectively reduces the divergence at the endpoints of the IMFs and yields physically meaningful IMF components. Finally, the method was applied to the analysis of actual blasting seismic signals. It successfully suppressed the endpoint effects of EMD and improved the extraction of time–frequency characteristics from blasting-induced seismic waves. This has significant practical implications for safety assessments of existing structures in areas affected by blasting.

## 1. Introduction

The analysis of blasting-induced seismic waves presents unique challenges due to their inherently non-stationary and nonlinear characteristics [1,2,3,4,5,6]. These transient signals convey critical information regarding vibration propagation and potential structural impacts. Empirical mode decomposition (EMD) [7,8,9,10,11,12] is based on the temporal scale characteristics inherent to the data, and the decomposition process preserves these characteristics. EMD, coupled with the Hilbert transform (EMD-Hilbert), has emerged as a particularly effective approach for analyzing complex waveforms [13,14,15]. The EMD method adaptively decomposes the blasting-induced seismic signal into a series of intrinsic mode functions (IMFs) [16,17,18,19,20], which represent the various oscillatory modes embedded within the original signal. Each IMF satisfies specific conditions that enable meaningful instantaneous frequency computation through subsequent Hilbert transform [21,22]. This combined approach provides a powerful time–frequency-energy representation that captures the evolving spectral characteristics of blast vibrations, which is essential for accurate hazard assessment and vibration control [23,24].

The EMD process decomposes complex blasting-induced seismic signals into a finite set of IMFs through an iterative sifting procedure. Each IMF must satisfy two fundamental conditions: (1) the number of extrema and zero-crossings must either be equal or differ by at most one, and (2) the mean value of the envelope defined by the local maxima and minima must be zero. These conditions enable subsequent meaningful instantaneous frequency computation through the Hilbert transform. The EMD-Hilbert methodology offers several distinct advantages for seismic analysis of blasts: firstly, its adaptive nature allows for the automatic capture of various vibration modes present in blast waves, eliminating the need for predetermined basis functions. Second, the resulting Hilbert spectrum provides a simultaneous high-resolution representation in time, frequency, and energy, revealing how various frequency components evolve throughout the blast event. Third, the method can identify nonlinear wave propagation effects and modal coupling phenomena that might be overlooked by conventional spectral analysis. These capabilities make EMD-Hilbert particularly valuable for precise hazard assessment, vibration control, and structural response prediction in blasting operations.

Despite its theoretical advantages, the practical implementation of EMD-Hilbert analysis for blasting-induced seismic waves encounters significant challenges due to the well-documented endpoint effect [25,26,27,28,29]. This inherent limitation of the EMD algorithm manifests during the sifting process, where cubic spline interpolation at signal boundaries causes IMF components to diverge. The distortion originates at both ends of the signal and progressively contaminates inward through the entire data series, with the severity of contamination being inversely proportional to the signal length. For typical blasting seismic records, which often consist of relatively short duration measurements, the endpoint effect can corrupt a significant portion of the decomposed signal. When these distorted IMFs undergo Hilbert transform, the resulting time–frequency representation contains artificial components that lack physical meaning, severely compromising the analysis’s reliability. In engineering practice, this is evident in inaccurate predictions of vibration frequencies and incorrect estimates of energy distribution, which can result in either excessive safety margins that raise operational costs or perilous underestimations of blast impacts.

The energy conservation principle in EMD provides a theoretical framework for understanding and addressing the endpoint effect. Ideally, the IMF components obtained through EMD should be approximately orthogonal, ensuring that the total signal energy remains invariant before and after decomposition, with minimal energy leakage between different IMFs. This orthogonality condition serves as a crucial evaluation metric for assessing the performance of algorithms that suppress endpoint effects.

Conventional methods for mitigating endpoint effects in earlier years, such as the mirror extension (ME), extreme value extension (EVE), or polynomial fitting (PF) [30,31,32,33,34,35], typically concentrate solely on local signal characteristics near the boundaries, often overlooking the global energy distribution and global waveform matching. These methods frequently fail to conserve energy appropriately throughout the entire signal duration, especially for the complex waveforms encountered in blasting seismology. In recent years, research on the endpoint effects of EMD has revealed several development trends. (1) To obtain ensemble empirical mode decomposition (EEMD), efforts have mitigated the endpoint effect to some extent; however, these improvements do not offer a fundamental solution to the issue. EEMD continues to suffer from the problem of endpoint divergence [36,37,38]. (2) There is a tendency to concentrate on the development of endpoints while overlooking the overall trends in the signal waveform [39]. (3) There is an emphasis on waveform matching, yet there is a disregard for the signals themselves [40].

To tackle these challenges, this study has developed an enhanced endpoint effect suppression algorithm that addresses four critical aspects: local endpoint development trends, global time distribution, energy matching, and waveform matching. By concentrating on local endpoint development trends, the algorithm precisely captures the dynamic changes of the signal at boundaries, thus preventing artificial distortion during decomposition. With a focus on global time distribution, it preserves the correct temporal sequence of various vibration components, ensuring precise time–frequency analysis. Energy matching is essential as it upholds the principle of energy conservation, avoiding energy leakage between the IMFs and enabling a reliable quantification of vibration energy for hazard assessment. Waveform matching, on the other hand, maintains the unique shape characteristics of blasting-induced seismic waves, allowing the decomposed IMFs to better reflect the complex dynamics of the waves. Collectively, these four aspects overcome the limitations of traditional methods, offering a more accurate, reliable, and effective approach for analyzing blasting-induced seismic waves using EMD.

To verify the algorithm’s effectiveness in suppressing EMD endpoint effects, comparative studies were conducted using simulated signals, comparing the proposed method with mirror extension, polynomial fitting, and extreme value extension methods. Three evaluation metrics were used: error standard deviation, correlation coefficient, and computation time. In the simulated signal experiments, the proposed algorithm demonstrated remarkable superiority. The error standard deviations for IMF1 and IMF2 decreased by 7.67–22.91% compared to conventional methods (ME, PF, EVE). The correlation coefficient between the decomposed IMF and the original signal’s true components reached above 0.95, far exceeding the results of the other methods, thus verifying the physical meaning of the obtained IMF components. Meanwhile, because the proposed EMD endpoint effect suppression algorithm takes into account four major factors, its computation is relatively complex compared to the other three methods. The computation time required is also greater than that of the other three algorithms.

Finally, the method was applied to the analysis of actual blast seismic signals. It successfully suppressed the endpoint effects of EMD and enhanced the extraction of time–frequency characteristics from blasting-induced seismic waves. In a case study involving a nearby building during a blasting operation, the algorithm accurately identified the dominant vibration frequencies and their energy distribution, providing reliable data for safety assessments of existing structures in blast-affected areas, thereby highlighting its significant practical value [41,42,43,44].

## 2. Method

### 2.1. EMD Process and Endpoint Effect Mechanism

The empirical mode decomposition (EMD) method necessitates multiple sifting iterations to extract meaningful intrinsic mode functions (IMFs) from the original signal. The sifting process primarily involves calculating the local mean signal by constructing upper and lower envelopes. The upper envelope is created by connecting all local maxima using cubic spline interpolation, whereas the lower envelope links all local minima. However, this process inherently suffers from endpoint effects due to two fundamental issues: (1) signal endpoints cannot simultaneously serve as both maximum and minimum points, and (2) the endpoints are not necessarily extreme points by definition.

These limitations result in the constructed envelopes diverging at the boundaries during the spline interpolation process. As the sifting iterations progress, this divergence spreads inward, contaminating the entire signal and causing significant IMF distortion. In the analysis of blasting-induced seismic waves, where signals are typically of short duration and contain high-frequency components, this effect becomes particularly pronounced.

### 2.2. Proposed Endpoint Effect Suppression Algorithm

To address these challenges, we developed a novel algorithm for suppressing the endpoint effect in EMD that systematically considers four critical aspects.

(1) Local endpoint development trends
(2) Global time distribution
(3) Energy matching
(4) Waveform matching

The algorithm operates through two carefully designed processing stages.

#### 2.2.1. Stage 1: Generation of the Dual-Trend Characteristic Wave (DTCW)

The initial phase involves generating the Dual-Trend Characteristic Wave (DTCW). First, the intrinsic relationship between the global signal time and the time interval of the endpoints is examined to determine the extended time parameter. Subsequently, the amplitude variation trend of the endpoints is considered to obtain the DTCW.

(1) DTCW time parameter

**Figure 1:**
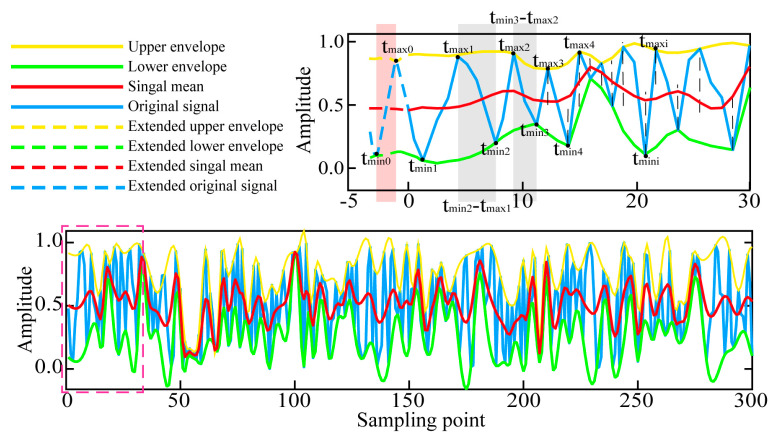
An example of DTCW extension process for the left endpoint.

Taking the left endpoint of the signal as an example, at the moment when all the maximum points of the signal are found, they are denoted as *t*_max1_, *t*_max2_, …, *t*_maxi_ (where *i* = 1, 2, 3… M). Similarly, the time when all minimum points of the signal are found to occur is denoted as *t*_min1_, *t*_min2_, …, *t*_mini_ (where *i* = 1, 2, 3…N). In these parameters, “M” represents the maximum number of points and “N” represents the minimum number of points. The maximum and minimum points to be extended are *t*_max0_ and *t*_min0_, respectively. The calculation can be divided into the following four cases. To facilitate understanding of the basic principle of the DTCW time parameter, a random signal with a length of 300 and amplitude ranging from 0 to 1 was established, as per Case 3. The DTCW time parameter for the left endpoint of the signal was then constructed, as illustrated in Figure 1.

Case 1: *t*_max1_ < *t*_min1_∩*t*_maxM_ < *t*_minN_, M = N, the solutions of *t*_min0_ and *t*_max0_ are shown in Equations (1) and (2), respectively.

(1) $$t_{\min0}=\frac{\sum_{i=1}^{N-1}\left(t_{\max i+1}-t_{\min i}\right)}{N-1}-t_{\max1}$$

(2) $$t_{\max0}=\frac{\sum_{i=1}^{N}\left(t_{\min i}-t_{\max i}\right)}{N}+t_{\min0}$$

Case 2: *t*_max1_ < *t*_min1_∩*t*_maxM_ < *t*_minN_, M = N + 1, the solutions of *t*_max0_ is the same as Equation (2), and the solution of *t*_min0_ is shown in Equation (3).

(3) $$t_{\min0}=\frac{\sum_{i=1}^{N}\left(t_{\max i+1}-t_{\min i}\right)}{N}-t_{\max1}$$

Case 3: *t*_max1_ > *t*_min1_∩*t*_maxM_ > *t*_minN_, M = N, the solutions of *t*_max0_ and *t*_min0_ are shown in Equations (4) and (5), respectively.

(4) $$t_{max0}=\frac{\sum_{i=1}^{N-1}\left(t_{\min i+1}-t_{\max i}\right)}{N-1}-t_{\min1}$$

(5) $$t_{\min0}=\frac{\sum_{i=1}^{N}\left(t_{\max i}-t_{\min i}\right)}{N}+t_{\max0}$$

Case 4: *t*_max1_ > *t*_min1_∩*t*_maxM_ < *t*_minN_. M = N − 1, the solution of *t*_max0_ is the same as Equation (4), and the solution of *t*_min0_ is shown in Equation (6).

(6) $$t_{\min0}=\frac{\sum_{i=1}^{N-1}\left(t_{\max i}-t_{\min i}\right)}{N-1}+t_{\max0}$$

(2) DTCW amplitude parameter

To identify the left signal boundary, we first locate all local maxima and record their values as *x*_max1_, *x*_max2_, …, *x*_maxi_ (where *i* ranges from 1 to M). Likewise, we find and denote the values of all signal minima as *x*_min1_, *x*_min2_, …, *x*_mini_ (wher*e i* ranges from 1 to N). Based on the trend of amplitude variation near the endpoint, we extend a maximum point and a minimum point close to the left endpoint. The specific steps are as follows:

Step 1: Obtain the 3–5 maximum value points closest to the left endpoint from the maximum value dataset.

Step 2: Fit these 3–5 maximum points and replace them with *t*_max0_ obtained in the previous step to solve for *x*_max0_, thus completing the extension of the maximum value of the left endpoint, i.e., (*t*_max0_, *x*_max0_). Similarly, it is possible to extend the minimum left endpoint and obtain (*t*_min0_, *x*_min0_).

#### 2.2.2. Stage 2: Dual-Criteria Matching Process

With the DTCW established, the algorithm performs energy and waveform matching.

(1) Adaptive energy matching

The orthogonality of the IMF serves as a crucial indicator to evaluate the inhibition effect of the EMD endpoint. When signal energy remains intact during the process of modal decomposition, and the IMF components do not overlap, with an orthogonal exponent of zero, the orthogonality is optimal. Consequently, by examining the energy correspondence between the DTCW and the entire signal, one can identify a “signal fragment” that exhibits the highest degree of correspondence with the DTCW. The specific steps are as follows:

Step 1: Utilize the left endpoint extension results acquired in Section 2.2.1, namely *(t*_max0_, *x*_max0_) and (*t*_min0_, *x*_min0_), along with the closest pair of maximum (small) value points (t_max1_, *x*_max1_) and (*t*_min1_, *x*_min1_), to construct a time series. This time series is designated as *S*(*t*)_L_ and comprises “m” sample points.

Step 2: Divide the original signal *S*(*t*) into “b” signal fragments, denoted as *S*(*t*)_1_, *S*(*t*)_2_, …, *S*(*t*)_b_, and calculate their contained energy *E*_1_, *E*_2_, …, *E*_b_.

Step 3: Calculate the energy *E*_L_ contained in *S*(*t*)_L_, and select an *E_k_*_1_ (1 ≤ *k*_1_ ≤ b) with the highest matching degree to *E*_L_. Equation (7) shows the calculation of the energy matching coefficient, *δ*_emc_, which is the energy difference between two “signal fragments.” When *δ*_emc_ is at its minimum value, the corresponding “signal fragment” *S*(t)_k1_ is the desired value. In the ideal state, *δ*_emc_ equals 0.

(7) $$\delta_{emc}=\left|E_{L}-E_{k1}\right|=\left|\int_{m}S^{2}{\left(t\right)}_{L}dt-\int_{m}S^{2}{\left(t\right)}_{k1}dt\right|$$

Let the original total energy of *S*(t) be *E*_s_, and the expression of *E*_s_ is shown in Equation (8).

(8) $$E_{S}=\int_{T}S^{2}(t)dt=\sum_{i=1}^{b}S^{2}{\left(t\right)}_{i}$$

Step 4: Move the *S*(t)_k1_ (1 ≤ *k*_1_ ≤ *b*) that satisfies the minimum value of *δ*_emc_ to the position of *S*(*t*)_L_ to obtain the signal processed by the endpoints.

(2) Adaptive waveform matching

The trend of a signal is reflected not only at its endpoints but also within the signal itself. This critical observation forms the basis for our waveform matching methodology.

*S*(*t*) = *S*(t)_1_ + *S*(t)_2_, …, + *S*(t)_b_. Taking the left endpoint *S*(*t*)_L_ as a reference, a “signal fragment” within *S*(*t*) that exhibits the highest matching degree (MD) with *S*(*t*)_L_ is identified, denoted as *S*(*t*)_k2_ (1 ≤ *k*_2_ ≤ *b)*.

The calculation of matching degree is shown in Equation (9), where *d* is the linear normalized result of the sum of distances between any two points on *S*(*t*)_L_ and *S*(*t*)_i_ (1 ≤ *i* ≤ *b*), as calculated in Equation (10). The correlation coefficient *r* is calculated by Equation (11). Since the development trend of the signal is reflected not only at the endpoints but also within the signal itself, it is evident that *r* > 0.

(9) $$\mathrm{MD}=\frac{d}{r}$$

(10) $$d=\frac{\left|\sum_{i=1}^{b}\left(S{\left(t\right)}_{L}-S{\left(t\right)}_{i}\right)\right|}{{\left|\sum_{i=1}^{b}\left(S{\left(t\right)}_{L}-S{\left(t\right)}_{i}\right)\right|}_{\max}}$$

(11) $$r=\frac{\mathrm{cov}\left(S{\left(t\right)}_{L},S{\left(t\right)}_{i}\right)}{\sigma \left[S{\left(t\right)}_{L}\right]\cdot \sigma \left[S{\left(t\right)}_{i}\right]}\begin{array}{cc} & \left(1\leq i\leq b\right) \end{array}$$

To more accurately quantify the degree of match between two “signal fragments,” the matching degree formula (Equation (9)) is introduced. This formula takes into account two key metrics: amplitude and waveform similarity. A smaller “*d*” indicates that the amplitudes of the two signals are more similar, whereas a larger “*r*” signifies a greater similarity between the waveforms. Consequently, for the MD, a lower value indicates a higher degree of match between the two signals, and vice versa. Our objective is to identify the *S*(*t*)_k2_ that has the highest degree of match with *S*(*t*)_L_.

Under normal conditions, the “signal fragment” *S*(t)_k1_ obtained through energy matching and the “signal fragment” *S(*(*t*)_k2_ obtained through waveform matching are identical, that is, *S*(t)_k1_ = *S(*(*t*)_k2_. The *S*(t)_k1_ = *S(*(*t*)_k2_ obtained from the left endpoint is the *S(*(*t*)_i_ proposed in the abstract. This convergence confirms the robustness and physical consistency of the proposed four-parameter-based endpoint effect suppression algorithm (considering global time variation, endpoint amplitude trends, energy matching, and waveform matching).

**Figure 2:**
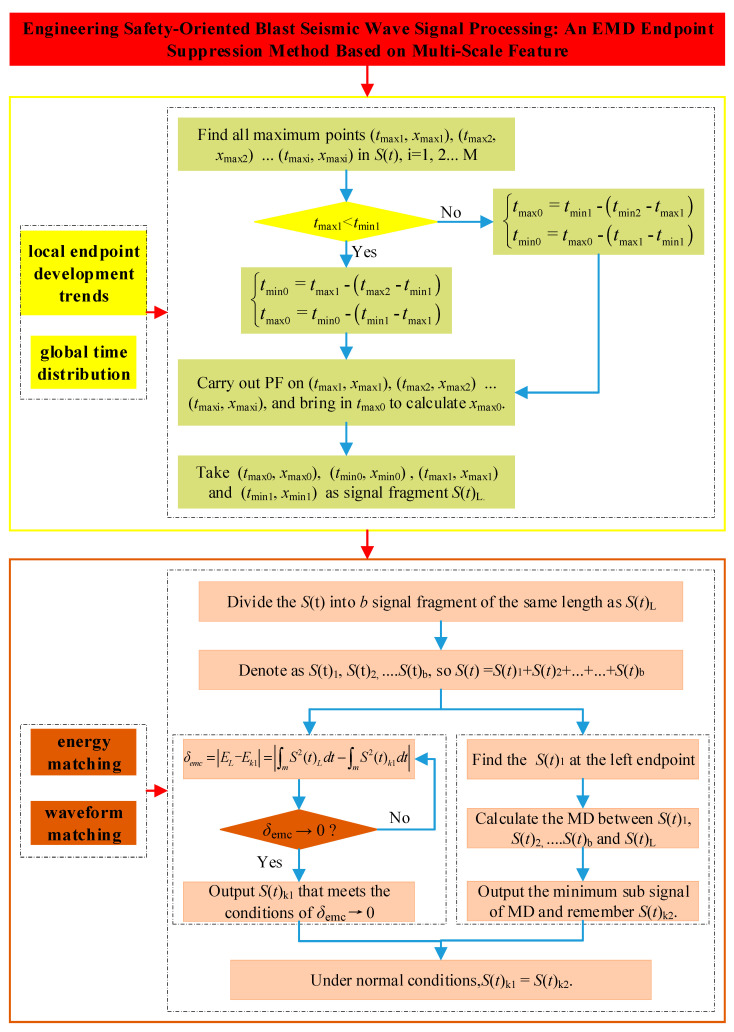
An EMD endpoint suppression method based on multi-scale feature.

To enhance understanding of the proposed EMD endpoint effect suppression algorithm, a flowchart illustrating the algorithm’s operation is provided in Figure 2. It is important to note that Figure 2 specifically demonstrates the suppression of EMD endpoint effects at the left endpoint as an example.

## 3. Comparative Study on Various Suppression Methods of Endpoint Effects in Simulation Signals

### 3.1. Generation of Simulated Vibration Signals

Simulation signals were used to conduct a comparative study on the suppression of various EMD endpoint effects. Subsequently, the suppression of the EMD endpoint effect was evaluated by combining the correlation coefficient, error standard deviation, and orthogonality.

Simulation signal *S*(*t*) = *x*_1_(*t*) + *x*_2_(*t*), namely *x*_1_(*t*) = sin (2 × π × 60 × t); *x*_2_(*t*) = (1 + 0.1 × sin (2 × π × 7 × t) × cos (2 × π × 30 × t). The time setting is as follows: sampling frequency *f*_s_ = 1000, sampling point N = 250, time t = (0: N − 1) × 1/*f*_s_.

**Figure 3:**
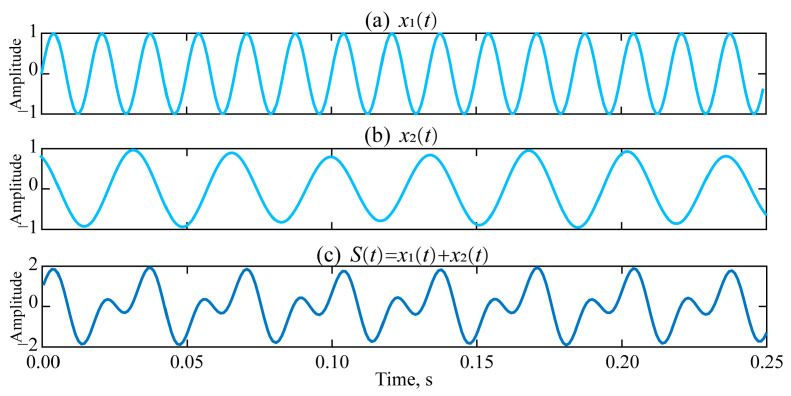
Simulation signal and its composition signal.

The waveform diagram of the simulated vibration signal is presented in Figure 3.

### 3.2. Simulation Vibration Signal EMD Endpoint Effect Suppression Processing

**Figure 4:**
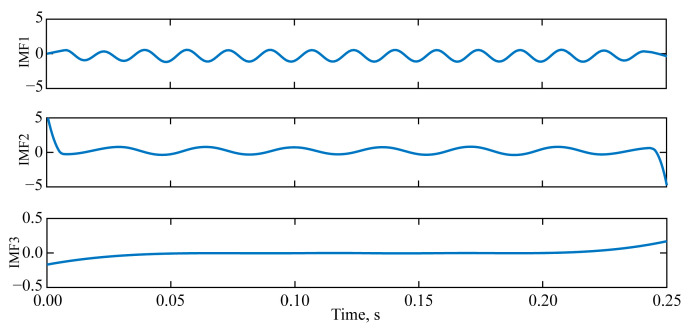
IMF component obtained without EMD endpoint effect suppression.

Upon examining Figure 4, it becomes evident that three IMFs were extracted without applying any suppression treatment for the end effects of the EMD. These IMFs are arranged in order of decreasing frequency. Both IMF1 and IMF2 display pronounced endpoint divergence. The frequency at the left and right endpoints of IMF1 tends to decrease from high to low frequency. Conversely, the frequency at the endpoints of IMF2 tends to increase from low to high frequency. Additionally, IMF2’s endpoints exhibit a notable jump in amplitude, where the amplitude deviates from the initial trend of development.

It is not difficult to observe that, when endpoint effects are not taken into account, the frequencies and amplitudes of IMF1 and *x*_1_(*t*) are similar, as are the frequencies and amplitudes of IMF2 and *x*_2_(*t*), which demonstrates that EMD is an adaptive algorithm. However, the presence of endpoint effects can cause distortion of the IMFs at the left and right boundaries. To recover the true vibration signal, it is necessary to suppress the EMD endpoint effects.

**Figure 5:**
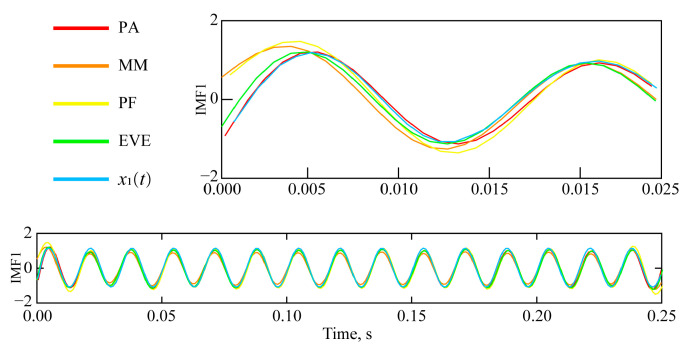
Comparison of IMF1 and *x*_1_(*t*) with different endpoint effect suppression algorithms.

In the following figure, we compare the IMF1 obtained by the algorithm proposed in the manuscript, the endpoint mirror extension, the polynomial fitting, and the extreme value extension method in the same graph, distinguishing them by different colors. The proposed algorithm (PA) uses red, the endpoint mirror extension (ME) uses orange, the polynomial fitting (PF) uses yellow, and the extreme value extension (EVE) method uses green. To highlight the contrast, *x*_1_(*t*) is also included in the IMF1 category and represented in blue. The detailed results are shown in Figure 5.

**Figure 6:**
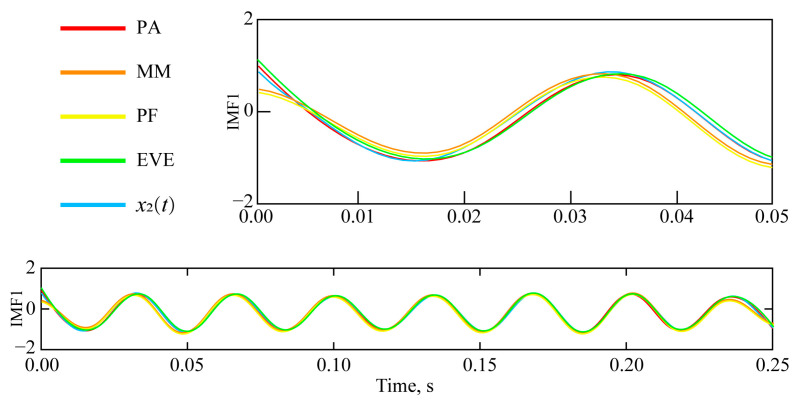
Comparison of IMF2 and *x*_2_(*t*) with different endpoint effect suppression algorithms.

Further comparison of IMF2 and *x*_2_(t), obtained by various endpoint effect suppression algorithms, is depicted in Figure 6.

### 3.3. Analysis of the Suppression Results of the EMD Endpoint Effect on Simulated Blasting Vibration Signals

The analysis of the suppression results of EMD endpoint effects by different endpoint effect suppression algorithms is conducted from two aspects. The first aspect is qualitative analysis, combined with the comparison between the IMF and component signals (*x*_1_(*t*) and *x*_2_(*t*)) obtained by different endpoint effect suppression algorithms shown in Figure 5 and Figure 6, to analyze the suppression effect of different endpoint effect suppression algorithms. The second aspect is quantitative analysis, combined with specific quantitative analysis indicators (correlation coefficient, standard deviation of error, and running time), highlighting the advantages of the proposed endpoint effect suppression algorithm.

The correlation coefficient (*r*) and the standard deviation of error (*D*_sde_) between the IMF and the corresponding sinusoidal signal were calculated individually, as was the running time of the endpoint effect suppression algorithm. The formula for *r* is presented in Equation (11), and the formula for *D*_sde_ is presented in Equation (12). The calculation results are displayed in Table 1.

(12) $$D_{sde}=\sqrt{\frac{\sum_{i=1}^{2}{\sum_{k=1}^{2}\left[\left(x_{i}\left(t\right)-IMF_{k}\left(t\right)\right)\right]}^{2}}{N}}$$

The following conclusions can be drawn from Figure 5 and Figure 6, and Table 1:

(1) Referring to the left endpoint of Figure 5, the divergence of IMF1 at the endpoint has been effectively suppressed. The suppression effects, from highest to lowest, are: PA > EVE > PF > ME.

(2) Referring to the left endpoint of Figure 6, the endpoint divergence of IMF2 has been effectively suppressed. The suppression effects, from most to least effective, are: PA > EVE > ME > PF.

(3) The endpoint suppression results and parameter calculation results of Figure 5 and Figure 6 show consistency.

① The correlation coefficients between IMF1 and *x*_1_(*t*), ranked from largest to smallest, are as follows: PA > EVE > PF > ME. PA achieved the highest correlation coefficient between IMF1 and *x*_1_(*t*), which increased by 16.56%, 15.41%, and 7.58% compared to ME, PF, and EVE, respectively.

② The error standard deviations between IMF1 and *x*_1_(*t*), from smallest to largest, are: PA < EVE < PF < ME. PA achieved the *D*_sde_ between IMF1 and *x*_1_(*t*), which decreased by 22.91%, 22.01%, and 13.03% compared to ME, PF, and EVE, respectively.

③ The correlation coefficients between IMF2 and *x*_2_(*t*), from large to small, are: PA > EVE > ME > PF. PA obtained the *r* between IMF2 and *x*_2_(*t*), which increased by 10.03%, 10.52%, and 5.52% compared to ME, PF, and EVE, respectively.

④ The error standard deviations between IMF2 and *x*_2_(*t*), from smallest to largest, are: PA < EVE < ME < PF. PA achieved the *D*_sde_ between IMF2 and *x*_2_(*t*), which decreased by 11.86%, 12.87%, and 7.67% compared to ME, PF, and EVE, respectively.

(4) Regarding the computation time of various endpoint effect suppression algorithms, the duration, from shortest to longest, is as follows: PF < EVE < ME < PA.

In summary, it is not difficult to discern that the proposed algorithm comprehensively considers four major factors, and the resulting endpoint effect algorithm effectively suppresses the inherent endpoint effect of EMD.

## 4. Application of the EMD Endpoint Suppression Method Based on Multi-Scale Feature in Blasting-Induced Seismic Wave Signal Processing

### 4.1. Engineering Background

**Figure 7:**
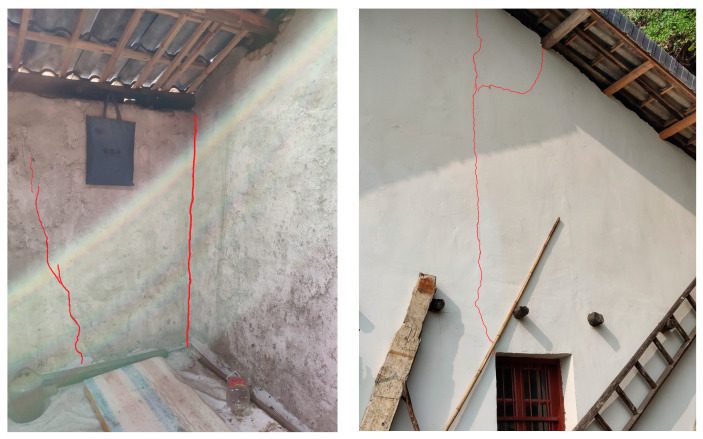
Various cracks caused by blasting construction.

The seismic wave test from real-world blasting data is based on a reef blasting project in Yichang. A private house located approximately 120 m from the blast zone was the primary focus of this monitoring effort. As a result of the blasting, numerous cracks emerged at the junctions of vertical and horizontal walls, as well as between the windows of the private house, as depicted in Figure 7. Consequently, the impact of blasting on nearby existing structures cannot be overlooked. Determining how to effectively manage the adverse effects of blasting is the main objective in the study of blasting hazards. An accurate analysis of the energy-frequency parameters that blasting-induced seismic waves impose on private houses is an essential step in the assessment of blasting hazards.

**Figure 8:**
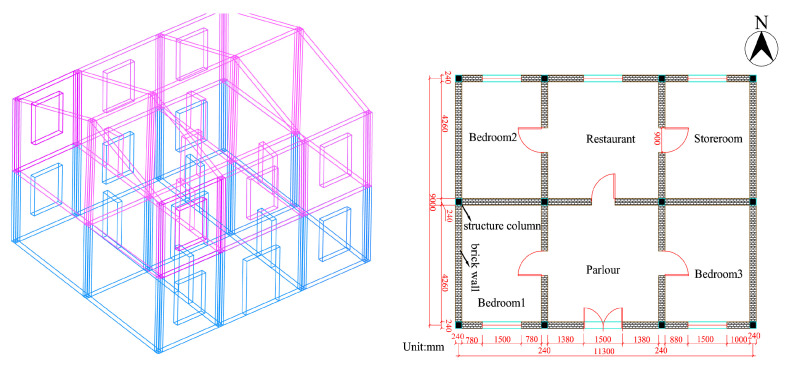
Schematic diagram of private house modelling.

To obtain the real and effective time–frequency characteristic parameters of a blasting-induced seismic wave, the monitored seismic wave signal must be processed at the endpoint. Only by doing so can the processed signal effectively suppress the endpoint effect of EMD, ensuring that the IMFs have practical physical significance. Figure 8 depicts a schematic of a private house, which is also the object of protection for this construction project.

### 4.2. Construction of the Model for Blasting-Induced Seismic Wave Signals

**Figure 9:**
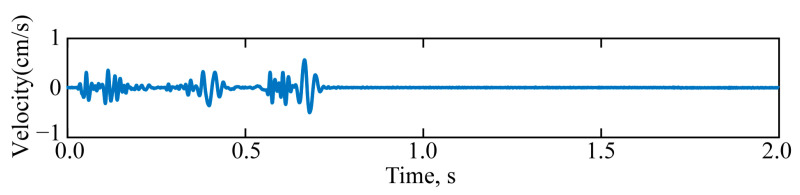
Seismic wave monitoring signal.

A typical seismic wave signal is selected from the monitoring data as the research subject, as depicted in Figure 9. The signal’s sampling frequency was 4000 samples per second (sps).

**Figure 10:**
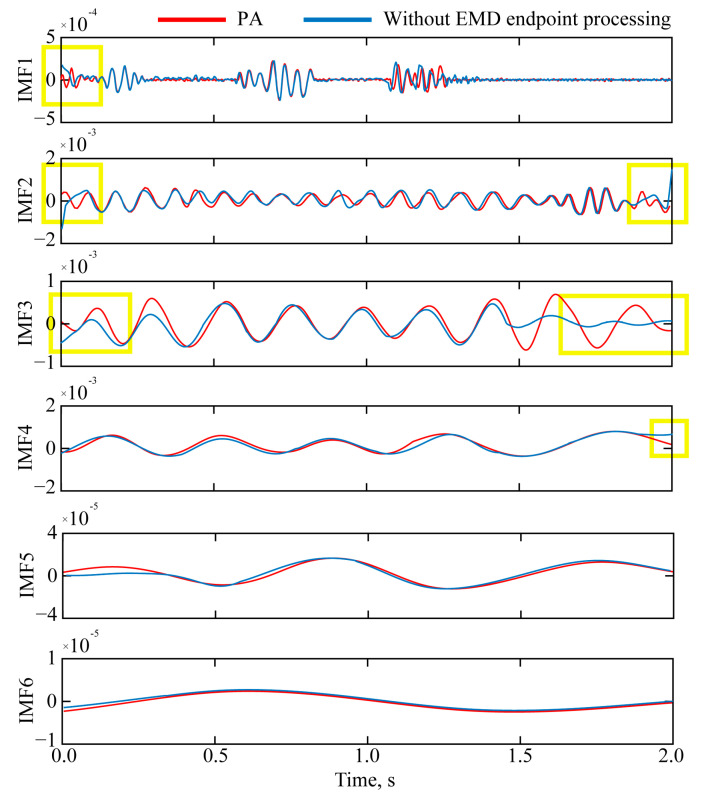
Comparison of proposed algorithm and IMF without EMD endpoint effect suppression.

Figure 10 compares the IMFs derived from the blasting-induced seismic wave signal. The red curves represent the results processed by the proposed EMD endpoint effect suppression algorithm, while the blue curves depict the raw IMFs without endpoint correction. The key observations and implications are as follows:

(1) The blue curves display significant endpoint divergence, especially in the mid-to-high-frequency components (for instance, the left endpoint of IMF1, and the left and right endpoints of IMF2 and IMF3). This appears as artificial amplitude amplification and frequency distortion near the signal boundaries, contravening the IMF’s principle of local symmetry.

(2) The proposed algorithm (red curves) effectively suppresses endpoint distortions, yielding IMFs with enhanced physical coherence. For instance, IMF1 (the highest frequency) now accurately captures transient blasting vibrations without edge contamination. The processed IMFs follow a strict frequency-descending order (IMF1 → IMF6), each representing a distinct octave of the signal’s energy.

**Figure 11:**
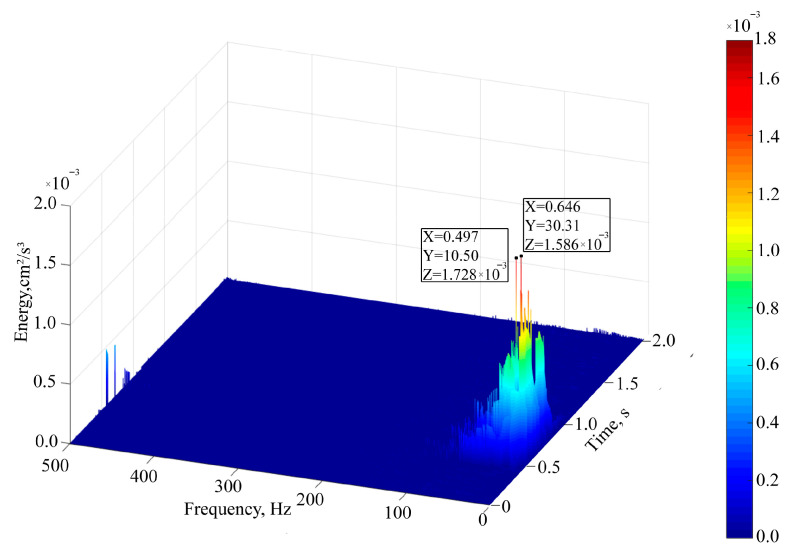
Three-dimensional time–frequency energy diagram of blasting-induced seismic wave signal.

Further analysis reveals that the IMF acquired through the endpoint effect suppression algorithm proposed in Figure 10 undergoes a normalized Hilbert transform (NHT) to yield a three-dimensional time–frequency energy graph of the entire signal, as depicted in Figure 11. It can be observed that the main frequency of the signal is 10.50 Hz and the secondary frequency is 30.31 Hz. The energy of the blasting-induced seismic wave is mainly concentrated in the low frequency range below 50 Hz. When the frequency of the seismic wave is the same as the natural frequency of the house, the amplitude of the structure will reach the maximum, thus inducing resonance harm.

### 4.3. Analysis of Results

The forced vibration of the object is carried out according to a certain specific law, that is, it has different natural frequency, which is because of the difference of its shape and structure.

To explore the natural frequency of the building structure, it is essential to analyze its vibration characteristics, including the characteristic frequency of vibration and the corresponding vibration modes, which is known as mode analysis. The mode analysis for the two-story house closest to the explosion zone has been conducted. The natural frequencies for each mode are presented in Table 2.

Analysis of Table 2 reveals that the fourth-order formation of the protected residential buildings is 9.68 Hz, and the main frequency of this blasting is 10.50 Hz, which is relatively close. Therefore, the seismic waves generated by this blasting construction may cause resonance in the residential building, and corresponding control measures must be taken in actual construction to ensure the safety of the residential building.

Further quantitative analysis is made on the influence of different IMF components on each formation of residential houses. It is not difficult to observe from Figure 10 that IMF1–IMF4 are the components with the highest energy proportion in this blasting. NHT was performed on IMF1, IMF2, IMF3, and IMF4 obtained by the proposed EMD endpoint effect suppression algorithm, and the main frequencies of a single IMF were obtained, which were 42.43 Hz, 30.65 Hz, 21.83 Hz, and 10.45 Hz, respectively.

The IMF has different amplification factors for different building structures. According to Equation (13), the amplification factors of IMF components on each formation of residential house can be calculated. The calculation results are shown in Table 3.

(13) $$D=\frac{1}{\sqrt{{\left(1-\beta^{2}\right)}^{2}+{\left(2\lambda \beta \right)}^{2}}}$$

In Equation (13), *β* represents the ratio of the dominant frequency of the IMF to the natural frequency of the house, while λ denotes the damping ratio. For the average building, λ typically takes a value of 0.05.

Upon examining Table 3, it becomes evident that as the natural frequency of the house approaches the primary frequency of the signal, the amplification factor increases. IMF4 can make the particle velocity of the 4th order array of residential houses produce 5.062 times amplification effect. When the main frequency of IMF is equal to the natural vibration frequency of the formation, D = 10, and the structural vibration amplitude reaches the maximum, which explains why the peak velocity measured at the residential building is only 0.54 cm/s, but there are still a large number of cracks in the structure caused by nearby blasting. The amplification factor of the IMF component varies for each type of residential building formation. The actual structure exhibits amplification characteristics under the influence of resonance. This factor is crucial to the cracking in residential houses. Consequently, in practical work, safety assessments cannot rely solely on a single speed peak as the criterion.

Based on the analysis presented, it appears that the seismic waves produced by the blasting could very well induce resonance in the private house. Consequently, it is essential to implement appropriate control measures during actual construction to guarantee the safety of the residential structure. In response to this phenomenon, it is recommended that subsequent construction adopt frequency increasing measures such as reducing the single stage charge amount, delaying detonation, optimizing the charge structure, or weakening the intensity of blasting-induced seismic waves by setting vibration reduction holes and grooves.

As comprehensively analyzed above, the suppression of the endpoint effect in EMD may seem like a minor aspect within the EMD-Hilbert transform framework. However, it exerts a profound and often underestimated influence on the accuracy of time–frequency analysis for blasting-induced seismic wave signals. This influence stems from the fundamental nature of EMD, a data-driven signal decomposition method based on the local characteristic time scale of the signal. When applied to blasting-induced seismic wave signals, which are inherently non-stationary and complex, the endpoint effect becomes a critical factor that cannot be overlooked.

The proposed EMD endpoint suppression algorithm comprehensively integrates four critical parameters (local endpoint development trends, global time distribution, energy matching and waveform matching), to effectively mitigate boundary distortions in the EMD process. By dynamically balancing these factors during the sifting process, the algorithm significantly improves the physical validity and mathematical precision of extracted IMF. This enhancement ensures reliable time–frequency representations of blasting-induced seismic waves, where each IMF accurately preserves the signal’s energy-frequency characteristics. The resulting high-fidelity IMF components form a solid foundation for subsequent Hilbert spectral analysis, enabling precise identification of hazardous vibration frequencies and amplitudes. Ultimately, this method provides trustworthy technical support for both blast vibration hazard assessment and the development of targeted control measures in engineering practice.

## 5. Discussion

The comprehensive analysis presented in this study demonstrates the critical importance of suppressing endpoint effects in EMD-based analysis of blasting-induced seismic waves. Our findings reveal several significant insights that advance both methodological development and practical engineering applications.

**First**, the comparative analysis demonstrates the advantage of the proposed four-parameter algorithm in suppressing EMD endpoint effects. Quantitative evaluations reveal significant improvements: correlation coefficients for IMF1 and IMF2 increased by 5.52–16.56%, while error standard deviations decreased by 7.67–22.91% compared to conventional methods (ME, PF, EVE). The algorithm consistently outperformed existing approaches in both qualitative waveform preservation (Figure 5 and Figure 6) and quantitative metrics (Table 1), validating its robustness.

**Second**, the engineering case study uncovers a pivotal resonance risk: the alignment between the blast’s dominant frequency (10.50 Hz) and the building’s fourth-order natural frequency (9.68 Hz). Despite a low peak velocity (0.54 cm/s), IMF4’s 5.062× amplification effect (Table 3) explains the observed structural damage, challenging conventional reliance on time-domain metrics alone. This underscores the necessity of integrating endpoint-corrected IMF analysis into safety assessments to capture frequency-dependent hazards.

**Limitations and Constraints**: We acknowledge the following technical constraints of our approach:

1. Signal Quality Dependence: The method requires a minimum of 3–5 detectable extrema near endpoints (Section 2.2.1) for reliable performance.

2. Performance Boundaries: Effectiveness degrades for (a) excessively noisy signals and (b) overly short segments, where boundary effects dominate the signal content.

These limitations are offset by the method’s adaptability to typical blast vibration signals and its computational efficiency for field deployment.

**Finally**, the study integrates signal processing theory with practical blast control. It validates the algorithm’s effectiveness using both simulated and real-world data, providing a framework for targeted mitigation strategies, such as charge optimization and delay adjustment. Future research should investigate real-time implementation and explore a wider range of structural typologies, but the current findings already confirm endpoint suppression as essential for accurate blast vibration assessment and structural integrity management.

**Future Research Directions**: This study’s endpoint suppression technique demonstrates promising potential for integration with advanced decomposition methods, such as the complete ensemble empirical mode decomposition with adaptive noise (CEEMDAN). The hybridization of these methods could synergistically combine CEEMDAN’s noise-resilient ensemble strategy with our boundary correction approach, potentially enhancing noise immunity while preserving physical interpretability. Future work will systematically investigate the performance of this combined methodology in handling challenging blast signatures, with an emphasis on maintaining computational efficiency for field applications. The proposed integration represents a logical next step in developing more robust EMD-based tools for precision blast monitoring.

## 6. Conclusions

This study presents three key findings regarding the enhanced EMD endpoint effect suppression algorithm.

(1) The proposed method integrates four critical parameters—local endpoint development trends, global time distribution, energy matching, and waveform matching—to achieve comprehensive endpoint effect suppression. This multi-dimensional approach ensures superior preservation of signal characteristics while effectively eliminating boundary distortions, enabling more precise extraction of time–frequency features in blasting seismic analysis.
(2) Systematic comparisons with conventional methods demonstrate the algorithm’s superior performance. Quantitative assessments reveal significant improvements, with correlation coefficients enhanced and error standard deviations reduced compared to existing techniques. The method’s effectiveness is consistently validated across both simulated and real-world blasting signals.
(3) The high-quality IMFs obtained enable more accurate time–frequency-energy spectrum analysis through Hilbert transform. The algorithm successfully extracts critical frequency–energy information from blasting vibrations, particularly facilitating the identification of potential resonance risks between blasting-induced vibrations and structural natural frequencies.

These findings establish the four-parameter optimization algorithm as a robust solution for EMD endpoint effect suppression, offering both theoretical advancements and practical improvements for blasting vibration monitoring and safety assessment. The method’s enhanced accuracy in signal decomposition and frequency characterization provides a reliable foundation for engineering applications requiring precise vibration analysis.

## Figures and Tables

**Table 1:** Evaluation index of end effect suppression method.

Evaluation Index		PA	ME	PF	EVE
*r*	IMF1 & *x*_1_(*t*)	0.9682	0.8026	0.8141	0.8924
	IMF2 & *x*_2_(*t*)	0.9517	0.8514	0.8465	0.8965
*D*_sde_	IMF1 & *x*_1_(*t*)	0.0324	0.2615	0.2525	0.1627
	IMF2 & *x*_2_(*t*)	0.0857	0.2043	0.2144	0.1624
running time (s)		0.1476	0.0173	0.0118	0.0169

**Table 2:** Natural vibration frequency corresponding to the first 6 modes of the private house vibration (unit: Hz).

Mode of Vibration	1	2	3	4	5	6
Natural frequency (Hz)	1.84	3.43	6.05	9.68	13.75	18.39

**Table 3:** Amplification factor of IMF component to residential house particles under different formations (unit: Hz).

Natural Frequency (Hz)	IMF Component			
	IMF1 (42.43)	IMF2 (30.65)	IMF3 (21.83)	IMF4 (10.45)
1 (1.84)	0.002	0.004	0.007	0.032
2 (3.43)	0.006	0.013	0.025	0.121
3 (6.05)	0.021	0.041	0.083	0.502
4 (9.68)	0.055	0.111	0.244	**5.062**
5 (13.75)	0.117	0.252	0.654	2.330
6 (18.39)	0.231	0.560	2.347	1.472

## Data Availability

Data is contained within the article.
